# Items of Interest

**Published:** 1895-04

**Authors:** 


### ITEMS OF INTEREST.

An effort is being made to establish a chair of scientific horseshoeing with lectures on the anatomy of the horse's foot, etc., in conjunction with the new Lewis Institute that is to be established in Chicago.

Under a pending law now before the State Legislature of New York State it is proposed to create a State Veterinarian. We trust the veterinarians of New York State will not let this opportunity for official recognition in the councils of the State be lost.

We have just learned that Cornell is again to be favored with an appropriation of $100,000 for the prospective veterinary department at that institution. We feel like congratulating New York State on her liberality to the profession and to this very deserving university.

The March number of *Peterson's Magazine* contains a very interesting account of the growth of the University of Pennsylvania. It is profuse with illustrations, among which we note the lecture-room of the veterinary department and the canine hospital, the latter said to be the most complete and best-arranged in the world.

Illinois is said to lead among the States in the number of horses owned, with 1,366,000 to her credit. New Jersey at the foot of the list with 88,000. Values average from $26 per head in Texas to $74 in the New England States. Missouri with

265,000 mules ranks first at an average of $41 per head. New Jersey possesses 8000, averaging $85 per head.

Much difference of opinion prevails in New York State as to the wisdom of passing, in the present form, the law to enforce an examination of all patent and proprietary medicines before being allowed to be sold. This should be done in every State, and the public more fully secured from the dangers of these nostrums that live by skilful advertising, playing upon the ignorance and cupidity of an innocent public. It should be an easy matter to arrange these differences of opinion, as they seem to only exist as to the methods of procedure.

The disjointed condition of the present laws in New York State relative to infectious and contagious diseases of live stock can hardly be said to be what should be expected of the Empire State. Aside from its expensive aspect it is too slow, too dangerous, and utterly methodless. With a tuberculosis commission to consider only bovine tuberculosis; with glanders under the control of the State Board of Health, and other diseases under the control and management of the Commissioner of Agriculture, what could be expected but dissatisfaction, annoyance, and indifference? All these diseases should be under one management: a Veterinary Department of the State Board of Agriculture, or a Live Stock Sanitary Board. No better field of work offers itself than the cure of this evil by the N. Y. S. V. M. A.

